# Integrating large mammal behaviour and traffic flow to determine traversability of roads with heterogeneous traffic on a Central Indian Highway

**DOI:** 10.1038/s41598-020-75810-2

**Published:** 2020-11-03

**Authors:** Akanksha Saxena, Nilanjan Chatterjee, Asha Rajvanshi, Bilal Habib

**Affiliations:** Wildlife Institute of India, Chandrabani, Dehra Dun, 248001 India

**Keywords:** Ecology, Behavioural ecology, Biodiversity, Urban ecology

## Abstract

Roads impact wildlife in multiple ways, most conspicuous amongst which are animal-vehicle collisions (AVCs). Mitigation measures to reduce AVCs at the local scale are often centred on species-specific crossing zones and collision hotspots. However, at the road network scale, consideration of interactions among road, species and traffic characteristics influencing AVC occurrence is required to design effective mitigation strategies. We modelled traversability—the probability of an animal successfully crossing a road- across an Indian highway for six large mammal species under different scenarios of road and traffic characteristics. Among the study species, group-living and slow-moving animals had higher AVC probabilities that increased significantly with increasing traffic volume and proportions of heavy vehicles in the traffic flow. The risk of AVC was higher for species that were active near roadside habitat during peak traffic hours. Our approach could help identify roads that pose potential mortality risks to animals using empirical data on animal and traffic characteristics. Results suggest that regulating traffic volume and heterogeneity on existing road stretches could potentially reduce animal mortality and barrier effect. Mitigation on roads expected to carry heavy traffic loads passing through ecologically-sensitive areas should be prioritised to ensure traversability for animal communities.

## Introduction

The transportation infrastructure of a nation is vital for its social and economic growth, especially for a developing economy. However, the construction and operation of roads come at great costs to wildlife and forests dissected by linear infrastructure^1,2^. The most conspicuous impact of roads is wildlife-vehicle collisions, which is a major cause of decline in animal populations in human-dominated landscapes^3^. Road-related mortality affects animal populations more adversely than natural mortality since it is non-selective, and affects healthy and unhealthy individuals of a population equally^4,5^.

Roads also cause some species to respond by avoidance of habitat near high traffic roads at peak traffic hours^6,7^. This avoidance of roadside habitat could result in the road becoming a barrier to animal movement^8^. In addition to the risk of local species extinction^9^, mortality and barrier effects together alter wildlife movement^10,11^ leading to isolation of populations^12,13^. With the global road network growth projected at more than 60%^14^, and rampant increase in worldwide vehicle ownership^15^, these impacts are set to accelerate in magnitude.

Variations in roadkill rates among species have been attributed to various environmental and species-specific features such as age group or life history stage^16–19^, sex and diet^20,21^, and animal activity patterns^6,22^. Therefore these traits are critical for identifying species most vulnerable to mortality and barrier effects for effective mitigation. Environmental and landscape features such as food resource distribution and habitat type^20,21^, and road-related features like traffic volume, vehicle speed, road width and road type^21,23–27^ also affect roadkill rates. Large traffic volumes may reduce the frequency of attempted crossings^7,22^, leading to low roadkill numbers, and could also cause the road to become a physical barrier to animal movement^28–31^.

Mitigation of roadkill and barrier effects of rapidly expanding road networks requires identification of road sections that may cause animal mortalities and barriers to animal movement, and species most likely to be involved in AVCs^8^. Studies that take into account road, traffic and landscape characteristics along with species presence, activity and movement characteristics^8,25,32^ have been able to predict mortality and barrier hotspots across road networks. However, the interaction among risk factors contributing to roadkill and barrier effects to inform mitigation strategies is largely missing from such models. Moreover, mitigation for rapidly expanding road networks should also be informed by road and traffic characteristics such as road types, and projections of traffic growth^33^ and traffic composition or ‘heterogeneity’ that is the proportion of different vehicle types in a traffic flow^8^.

We present a framework to identify species and roads vulnerable to AVC as a function of road, traffic and species characteristics, using data from traffic simulations under different traffic heterogeneity and volume scenarios, and morphometry and behavioural data of six widespread large mammals of Central India. The study was conducted on a 60 km stretch of the National Highway 44 (NH 44) passing through the Pench Tiger Reserve (PTR) in Madhya Pradesh and Maharashtra states.

We sought to answer four questions: (1) which species traits increase AVC probability (defined as the probability of an animal colliding with a vehicle while crossing a road, and is to be interpreted as the opposite of traversability)? (2) Which road and traffic characteristics make roads less traversable (i.e. increase AVC probability) for animal movement? (3) At what traffic volumes and compositions do roads become barriers for animal movement? (4) Does animal activity near roads increase AVC risk (defined as the hourly AVC probability as a function of animal activity)?

We hypothesised that slow-moving and group-living animals would have greater AVC probabilities under fast-moving highly heterogeneous traffic conditions. Additionally, we hypothesised that animals with high activity overlaps with traffic would have greater AVC risk.

## Results

### Influence of species traits on AVC probability

**Figure 1:**
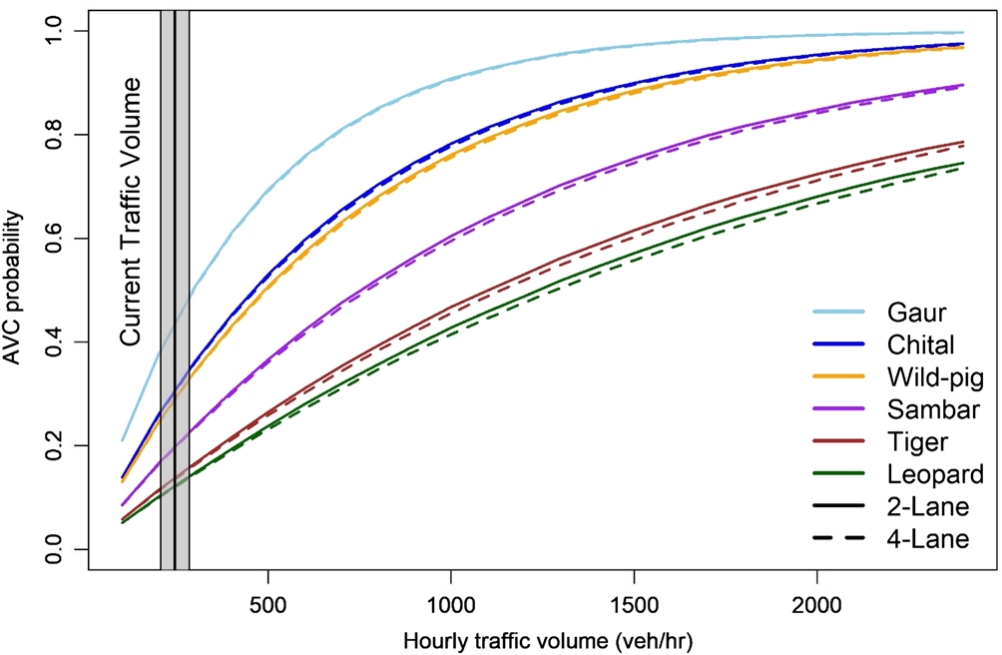
Animal Vehicle Collision probabilities with increasing hourly traffic volume (projected) at present traffic heterogeneity conditions on 2-lane (solid lines) and 4-lane (dashed lines) sections of the National Highway 44, passing through the Pench Tiger Reserve, India. Figure created using R^45^ version 3.6 (https://www.rstudio.com/).

Among the six study species, group-living species (gaur, chital, wild pig, sambar) had the highest AVC probabilities under hourly traffic volumes ranging between 100 and 2400 vehicles/h with present traffic heterogeneity (61% car, 13% bus/truck, and 26% MAVs) on NH44 (Fig. 1). Under present traffic heterogeneity and average hourly traffic volume (245 ± 20 vehicles/h) on NH44, lowest average daily AVC probabilities were found for tiger (0.13 ± 0.05) and leopard (0.11 ± 0.04) (Fig. 1). Chital (0.29 ± 0.10) and wild pig (0.29 ± 0.20) had similar average daily AVC probabilities, while sambar (0.42 ± 0.13) had a lower AVC probability among ungulates. Across both lane types, gaur had the highest average daily AVC probability.

**Figure 2:**
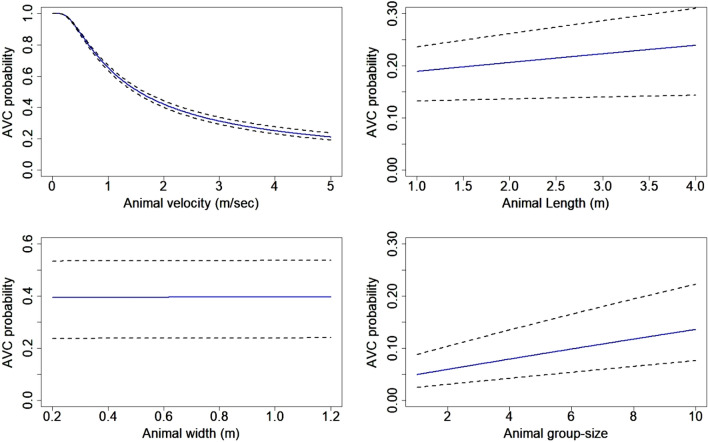
Relative effects of species traits on Animal Vehicle Collision (AVC) probability. To calculate the change in AVC probability with unit change in the target variables, the mean values of other variables were plugged into Eq. (1). Dotted lines indicate 95% confidence intervals around coefficient estimates. Figure created using R^45^ version 3.6 (https://www.rstudio.com/).

We found that an increase in group size increased the probability of hit (species length PIC 0.003; 95% CI 0.001–0.005) while increasing animal velocity decreased the probability of hit (PIC − 0.005; 95% CI − 0.011 to − 0.002). Animal width did not influence AVC probability significantly (PIC 0.04; 95% CI − 0.043–0.1) (Fig. 2).

### Influence of road and traffic characteristics on AVC probability

**Figure 3:**
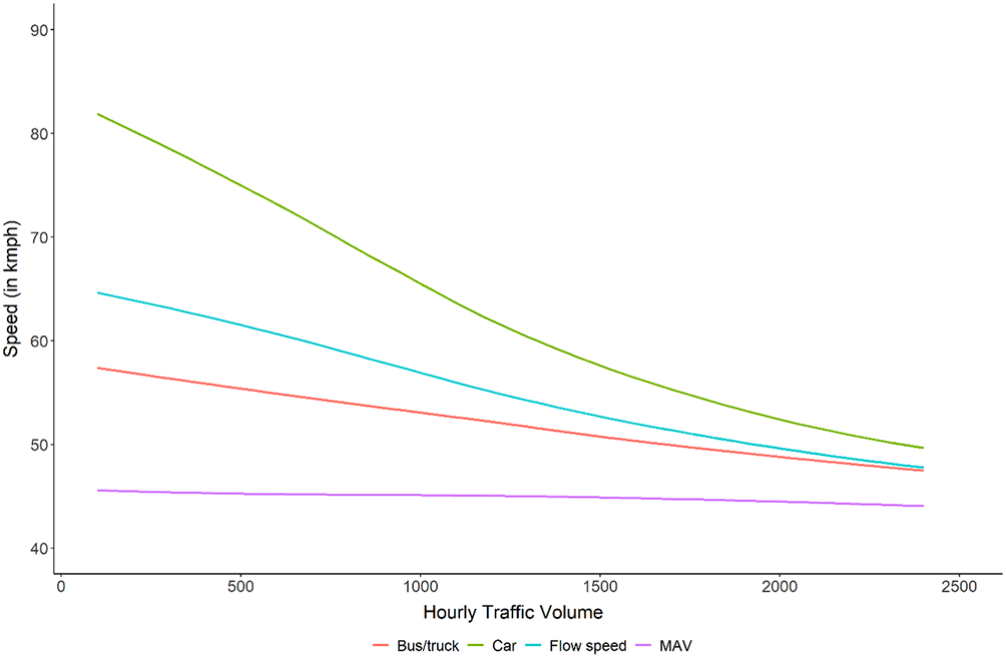
Simulated average harmonic traffic flow speed of present heterogeneity H_0_ (blue curve) on NH 44 at different traffic volumes, and free flow speeds of different traffic components viz., car (green), Bus/truck (red) and MAV (purple) in the same simulation. Figure created using R^45^ version 3.6 (https://www.rstudio.com/).

The average daily traffic volume on NH 44 during the study period was 5883 ± 168 vehicles, while the average hourly traffic volume was 245 ± 20 vehicles/h, comprising of 61% car, 13% bus/truck, and 26% MAVs. Simulations for present traffic heterogeneity on NH 44 at different hourly traffic volumes showed that heavy vehicles (MAVs) had the lowest, and lighter vehicles viz., cars had the highest free flow speeds within a traffic flow (Fig. 3).

For simulated heterogeneity scenarios at different hourly traffic volumes, the average harmonic traffic flow speeds of homogeneous traffic scenarios were higher than that of heterogeneous scenarios with greater proportions of heavy vehicles. Results of the traffic flow speed simulation under different heterogeneity scenarios have been provided in Supplementary Figure S1.

**Figure 4:**
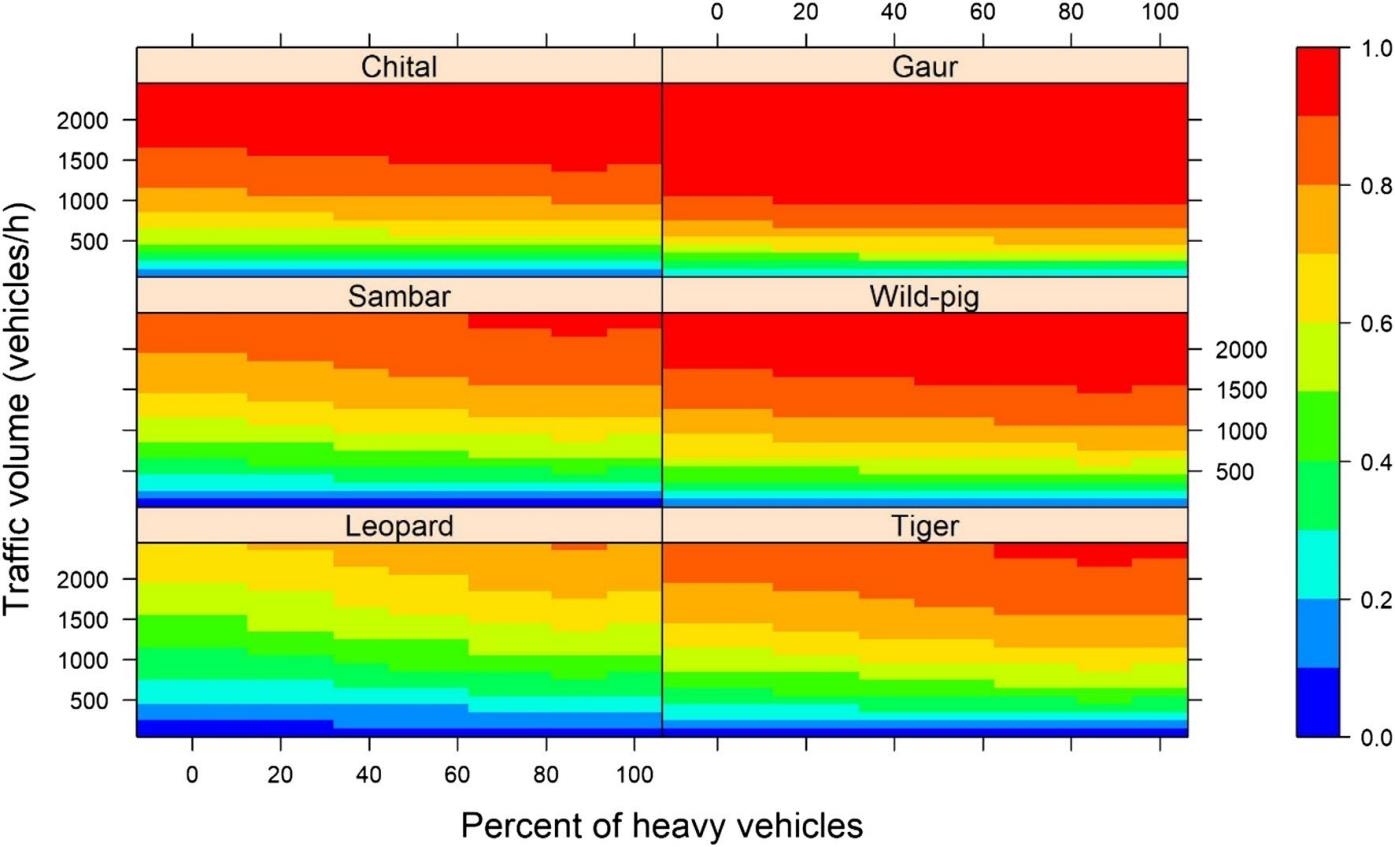
Gradient of Animal Vehicle Collision (AVC) probability for six study species across increasing traffic volumes under different percentages of heavy vehicles (representative of different heterogeneity scenarios). Colour bar to the right of plots depicts AVC probability (*P*_*h*_). Figure created using package ‘lattice’^58^ in R^45^ version 3.6 (https://www.rstudio.com/).

Across all simulated traffic heterogeneity scenarios, the AVC probability (*P*_*h*_) for all species increased with increase in traffic volume and showed similar variation among species as under present traffic heterogeneity. All animals had high *P*_*h*_ for traffic mostly comprising of heavy vehicles (scenarios H_3_, H_5_, H_6_) while low *P*_*h*_ was observed for scenarios where traffic mostly composed of light vehicles (H_0_, H_1_) (Fig. 4).

The threshold traffic volume at which AVC probability was equal to probability of successful crossing (*P*_*h*_ = 0.5) varied for different species. Under present traffic heterogeneity, this threshold traffic for group living animals like chital, gaur and wild pig was 300–400 vehicles/h. This threshold traffic volume was higher for sambar (700 vehicles/h), and highest for tiger and leopard (1100–1300 vehicles/h). Across all simulated scenarios, traffic volumes of 200–300 vehicles/h decreased the chances of a successful crossing by half for gaur. For chital, this traffic volume lies above 400–500 vehicles/h in traffic composed of light vehicles (H_0_, H_1_, H_4_, H_7_). This threshold volume is higher for sambar (1100 vehicles/h) in traffic composed mostly of heavy vehicles (H_3_, H_5_, H_6_). Hourly traffic volumes above 1000 vehicles/h posed a barrier to < 50% successful traverses for tiger and leopard at most traffic heterogeneity scenarios. The threshold traffic volume for solitary species was lowest (800 vehicles/h) at scenario H_3_ which comprised of only MAVs. Beyond this threshold traffic volume, higher fatalities are expected to occur.

**Figure 5:**
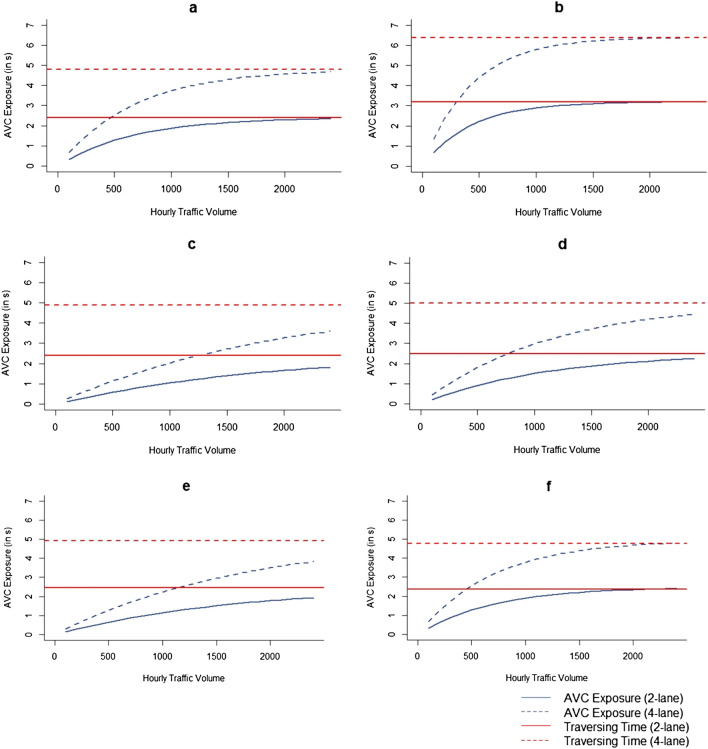
AVC exposure (blue curves; in seconds) of (**a**) chital, (**b**) gaur, (**c**) leopard, (**d**) sambar, (**e**) tiger, and (**f**) wild pig, a function of the duration of time an animal needs to traverse different widths of roads (red lines) and the Animal Vehicle Collision (AVC) probability under the hourly traffic volume at that time. Figure created using R^45^ version 3.6 (https://www.rstudio.com/).

For all study species, we found that the time on the road during which an animal is vulnerable to AVC (expressed as AVC exposure in seconds) was almost double (197.94% ± 1.72) on 4-lane segment as compared to 2-lane segment at the same traffic volumes (at present traffic heterogeneity) (Fig. 5). For chital, gaur and wild pig, AVC exposure at hourly volumes beyond 1300 vehicles/h almost reached asymptote i.e., the animal was at risk of collision for the entire traversing duration.

**Figure 6:**
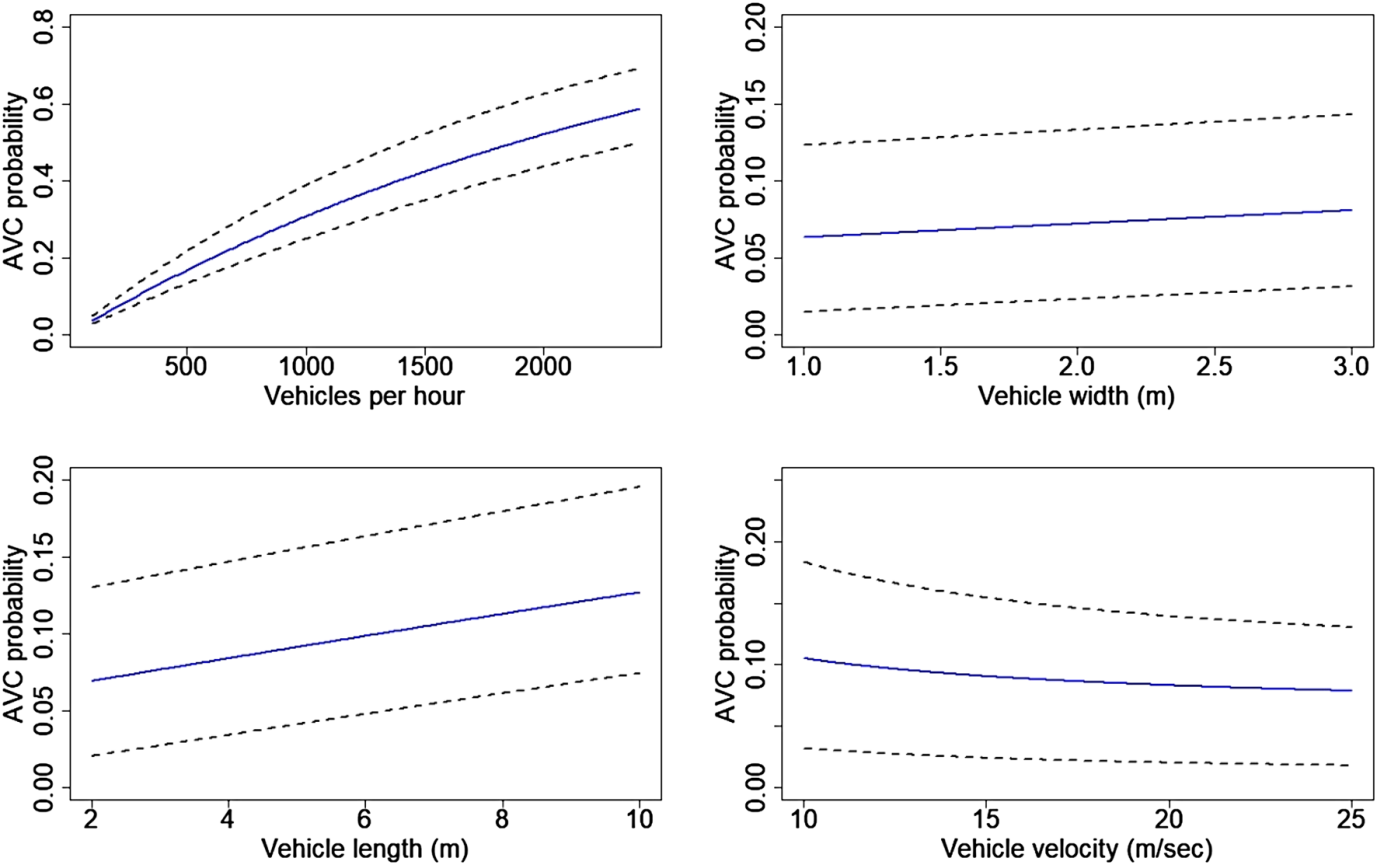
Relative effects of traffic characteristics on Animal Vehicle Collision (AVC) probability. To calculate the change in AVC probability with unit change in the target variables, the mean values of other variables were plugged into Eq. (1). Dotted lines indicate 95% confidence intervals around coefficient estimates. Figure created using R^45^ version 3.6 (https://www.rstudio.com/).

Change in traffic volume and traffic flow speeds had the strongest influence on *P*_*h*_ (Fig. 6). From the sensitivity analysis we found that AVC probability was positively influenced by length of the vehicle (PIC 0.032; 95% CI 0.02–0.039), and negatively influenced by velocity of vehicles (PIC − 0.093; 95% CI − 0.11 to − 0.07). We found no significant contribution of width of the vehicle (PIC − 0.015; 95% CI − 0.016–0.047). Traffic flow speeds decreased with increasing traffic volume and this change in speeds tended to increase *P*_*h*_ across all traffic heterogeneity scenarios on both lane types (Supplementary Fig. S1).

### Influence of animal activity on AVC risk

We photo-captured a total of 6043 photographs across 23 mammals, and 3624 images of our study species. The total sampling effort was 3024 camera-days at 216 camera trapping locations. Tiger and leopard were not considered for this analysis owing to low number of captures (n = 12 and n = 1 respectively) and larger home ranges as compared to the trapping area.

**Figure 7:**
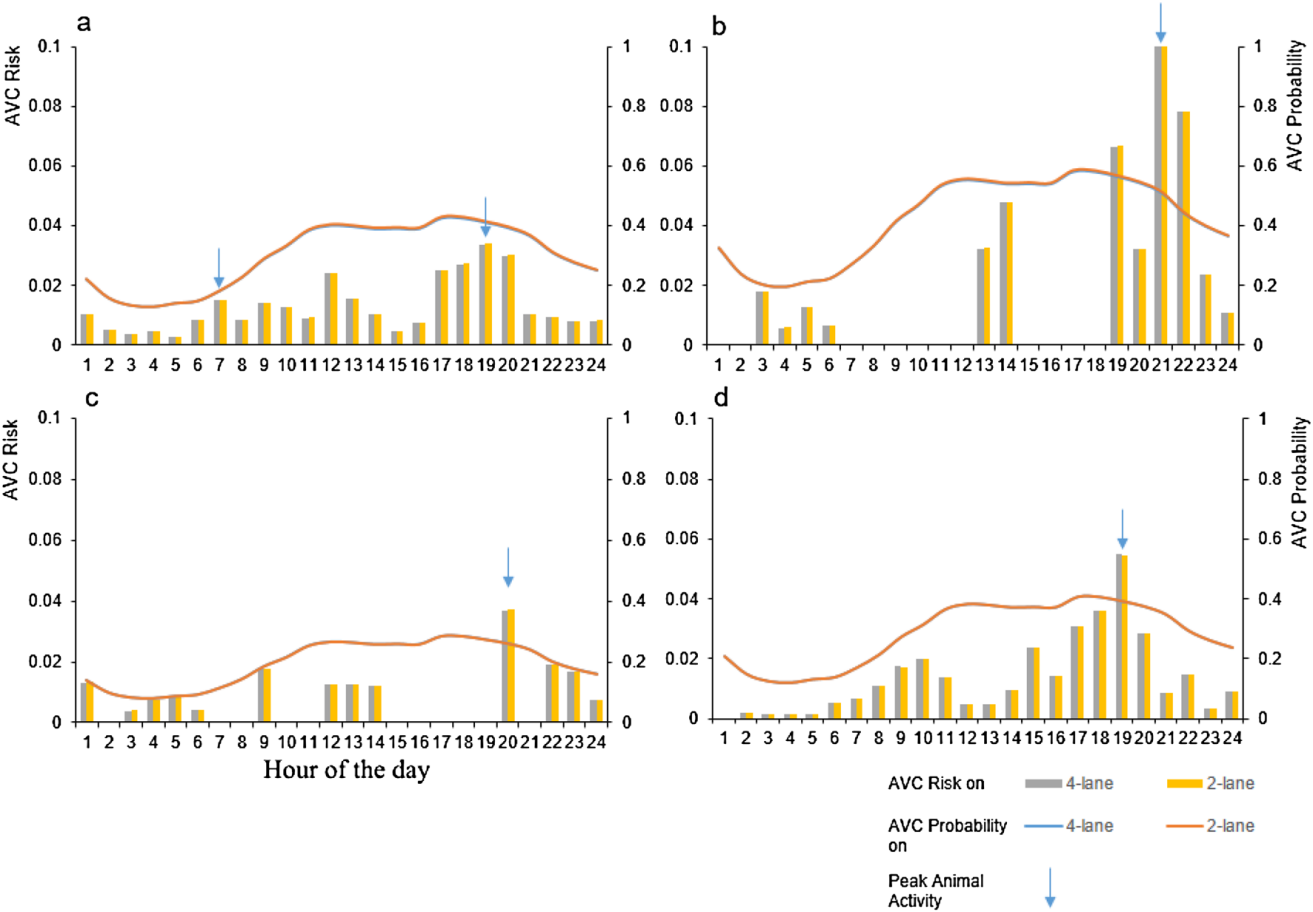
Animal Vehicle Collision (AVC) risk (yellow and grey bars) and AVC probability (red and blue curves) across 24 h for (**a**) chital, (**b**) gaur, (**c**) sambar and (**d**) wild pig. The AVC risk plotted on the secondary axis was computed as the product of AVC probability and hourly detection probability (used as a proxy for animal activity). Figure created using R^45^ version 3.6 (https://www.rstudio.com/).

We used species-specific hourly AVC probabilities and corresponding hourly detection probabilities calculated from captures of chital (n = 802), gaur (n = 34), sambar (n = 21) and wild pig (n = 79) to calculate hourly AVC risks for the four study species. We found differential effects of traffic and animal activity on AVC risk (Fig. 7). At peak traffic hours, AVC risk for wild pig was higher than for chital on both 2-lane and 4-lane roads. For sambar and gaur, there was no risk of AVC during peak traffic hour because their activities near the road did not coincide with traffic activity at peak traffic hour. The peak activity of chital during evening hours coincided with a traffic activity peak, and thus the AVC risk for chital was expected to be highest during this time, on both 2-lane and 4-lane road. Moreover, the activities of sambar and gaur showed lesser overlap with traffic activity while chital and wild pig had comparatively higher overlap (Supplementary Table S4).

## Discussion

We demonstrated the applicability of the traversability model to determine species-specific AVC probabilities and AVC risk across different traffic heterogeneity and traffic volume scenarios. We found that slow moving animals and animals with large group sizes were at higher risk of AVC as a consequence of greater time required to traverse roads. Further, AVC probability is expected to be greater on wider roads, particularly high traffic roads with predominantly heavy vehicles.

Among the six study species, lowest AVC probabilities were observed for tiger and leopard, primarily because these are solitary fast moving species. Body size had negligible effect on AVC probability^23^, but increase in group size increased the probability resulting in higher AVC probabilities for group living species. Among social species, lower group size of sambar translated to lower AVC probability than chital and wild pig, despite having similar maximum running speeds. Group size of gaur was similar to that of wild pig; yet gaur had the highest AVC probabilities across all heterogeneity scenarios as a result of its low running speed.

Mammals with low reproductive rates and high mobility, like carnivores show more negative responses to roads and traffic^34^, and carnivore movement is affected more than herbivores^35,36^ at high traffic volumes. Even though both carnivores in our study had the lowest AVC probabilities among all study species, they may not attempt crossing wider roads at high traffic volumes which could ultimately present a barrier to their movement^19^.

The AVC probability across different heterogeneity scenarios showed variability, largely as a consequence of the speeds of traffic flow. Results show that the highest AVC probabilities occurred for traffic compositions with higher proportions of heavy vehicles. Since heavy vehicles impede the cumulative traffic flow speed, the probability of occurrence of a vehicle at any point on the road increases, consequently translating to higher probabilities of hit mostly for slow-moving and group living animals. On the contrary, in a traffic flow with high flow speed, there is greater inter-vehicular distance available which translates to higher probability of an animal to cross the road without encountering a vehicle. This finding has implications for speed-regulating mitigation measures like speed breakers and rumble strips that can potentially decrease inter-vehicular distances at medium–high traffic volumes, leading to creation of a barrier-like situation for animal movement.

At higher traffic volumes the barrier effect sets in due to continuous flow of traffic, where road crossing by animals may not occur. Thus higher traffic volumes would result in a barrier-like situation because of avoidance of animals at high traffic segments^6,7^ reducing the number of attempted crossings by wildlife. This avoidance would reflect as low roadkill counts on high traffic roads^22^.

Even though AVC probabilities on wide road segments were similar to probabilities on narrow roads, the exposure of an individual/animal group to AVC while traversing a 4-lane segment was found to be almost double than the exposure on 2-lane segment. This risk of exposure was highest for species with large group sizes. Greater exposure on 4-lane roads at moderate traffic levels could cause more roadkill than on 2-lane roads, while on 2-lane roads the same traffic levels could become a barrier to animal movement.

The traversability model^37^ did not consider animal activity near roads. For an animal-vehicle collision to take place, an animal and a vehicle must co-occur on the road. We accounted for this by using animal activities near the road as a proxy for the probability of an animal encountering a road. Creation of edge habitats by linear intrusions like roads facilitate the use of such habitats by some ungulate species. Consequently for edge-tolerant species like chital and wild pig^38^ that were found to use road-forest edges, hourly roadkill risk is a function of hourly traffic volume since their activities near the road coincide with peak hours of traffic activity (Chital and Traffic Overlap Coefficient Dhat1 = 0.82; Wild pig and Traffic Overlap Coefficient Dhat1 = 0.82). Hence use of roadside habitat by chital and wild pig, makes them more vulnerable to mortality effects. For gaur and sambar that are generally crepuscular and nocturnal species with low road-forest edge use, the roadkill risk is a direct consequence of its activity in the early morning or late evening hours (Supplementary Table S4).

At present, traffic on NH 44 may not present a barrier to movement of chital and wild pig as we frequently encounter chital and wild pig roadkill at present traffic volumes. Gaur and sambar, on the other hand, are more vulnerable to barrier effect through avoidance behaviour of roadside habitat that reflects a lower tolerance to traffic disturbance^39,40^.

As in the aforementioned studies^8,23,37^, the aim of the study is not to determine the actual number of mortalities, but to help determine high-risk roads, traffic compositions and species groups most vulnerable to collision and barrier effects. Our model defines a way to identify components of a road network most vulnerable to roadkill, and could be used to identify road network components requiring prioritised mitigation action. In the absence of data on animal movement near roads, the present approach using species traits and behaviour could help prioritise mitigation for species most vulnerable to collision with vehicles.

Our framework can help inform mitigation of AVC and barrier effects in two ways: by identifying existing and proposed roads in a network that are or may become barriers to animal movement because of present and projected traffic volume, and by informing measures on existing roads with no structural mitigation measures based on traffic and animal activity. This is important for developing economies with rapidly increasing traffic loads on existing unmitigated road networks.

For existing road networks, our approach could help identify roads that are vulnerable to high roadkill rates as a function of their traffic volume and traffic composition. Measures may include speed restrictions for traffic predominantly consisting of small vehicles, temporal limitations on heavy vehicles and construction of animal passages on high traffic multiple use roads. Other actions such as alternate road network alignments considering projected traffic growth, combined with additional information on animal corridors, species occurrence and animal activity, can inform future road developments at the landscape scale.

Planning mitigation measures in a rapidly expanding road network with multiple use roads and highly heterogeneous traffic has to be futuristic and strategic i.e., it should consider large-scale and long-term conservation needs. Under such conditions, traffic volume is a critical parameter that would inform landscape-level prioritization with respect to present and projected traffic loads. Accommodating increased traffic on existing roads versus building new roads is an ongoing debate—while it has been stated that accommodating increased traffic growth on existing roads would be less damaging^33^, threshold traffic volumes may reduce animal road crossing rates, and create barrier to animal movement^39^. Thus, including traffic growth projections while planning mitigation for new roads (planning mitigation structure types, rerouting) would make mitigation viable for the long-term.

## Methods

### Study area

**Figure 8:**
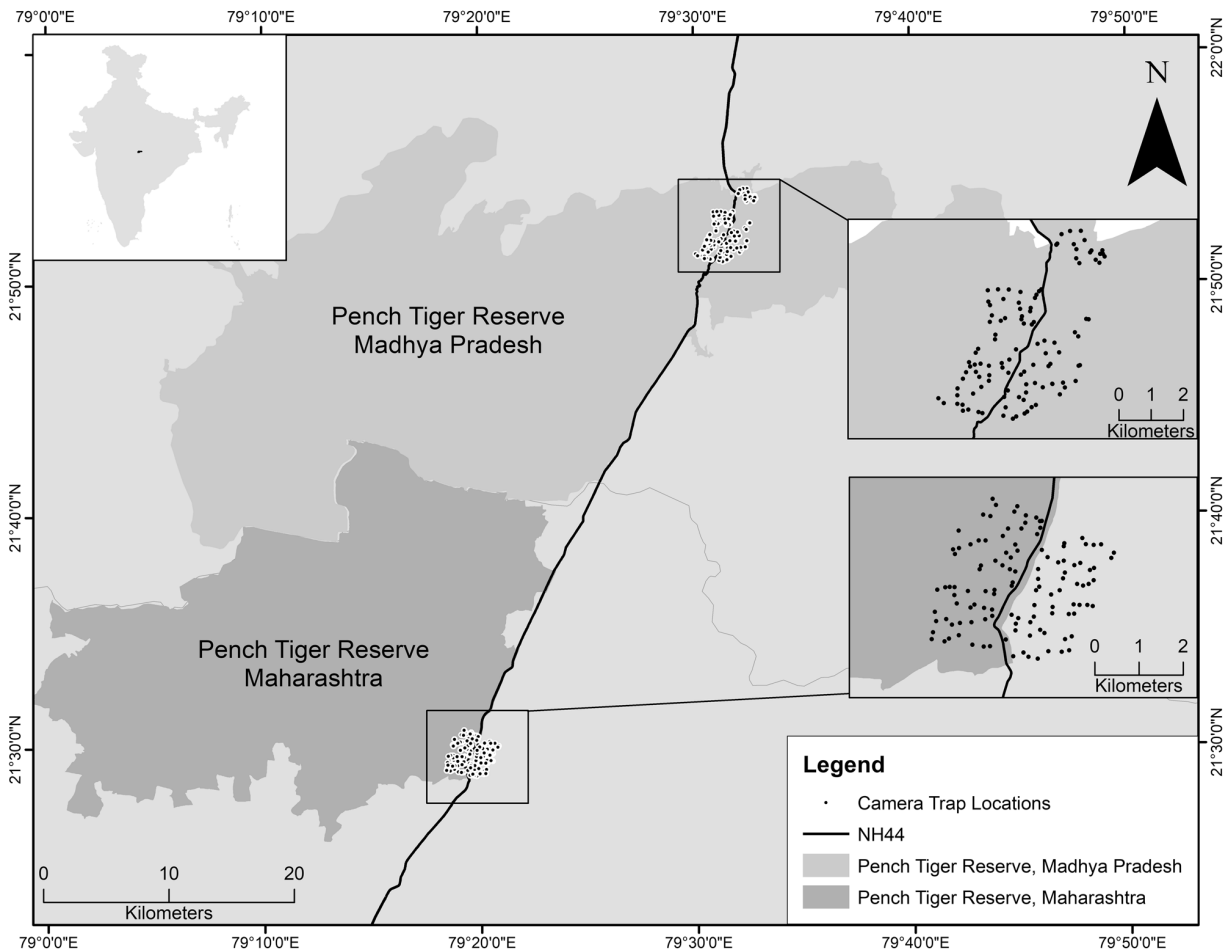
National Highway 44 passes through the Pench Tiger Reserves, Madhya Pradesh and Maharashtra.[Inset] Camera trapping locations on two forest stretches along NH 44. Map generated using ArcGIS 10.8^59^ (www.esri.com).

We conducted our study on a 60 km segment of the NH 44 (previously NH 7) that straddles the Maharashtra-Madhya Pradesh interstate boundary (Fig. 8) in central India. The highway forms the North–South transportation corridor, connecting major Indian economic and urban centres. The study was conducted on a 16 km 4-lane segment passing through Pench Tiger Reserve, Maharashtra, and a 29 km 2-lane segment passing through Pench Tiger Reserve, Madhya Pradesh. The tiger reserves are part of the Central Indian Landscape, which is a priority tiger conservation unit^41^. The forest in the area is mostly of the moist and dry deciduous type^42^. Tiger *Panthera tigris*, leopard *Panthera pardus*, sloth bear *Melursus ursinus* and wild dog *Cuon alpinus* are the major carnivores, and gaur *Bos gaurus*, chital or spotted deer *Axis axis*, sambar *Rucervus unicolor*, wild pig *Sus scrofa*, nilgai or blue bull *Boselaphus tragocamelus*, chausingha or four-horned antelope *Tetracerus quadricornis* are the major ungulate prey species found in the landscape. Tiger and leopard—the two apex predators, and gaur, spotted deer, sambar, and wild pig, common prey species of tiger and leopard were selected as the study species.

### Influence of species traits on AVC probability

We modelled the probabilities of successful crossing or hit for different animal species based on the traversability model^37^. The model is based on the calculation of headway distributions in a traffic flow, i.e., the frequency of the distance between successive vehicles at a given cross-section, which is assumed to follow Poisson distribution^43^. The probability of successful crossing (*P*_*a*_) depends on the time ‘*T*’ (in seconds) during which the animal and vehicle are co-incident on a road, and depends on traffic, road and species characteristics. Therefore,

1 $${P}_{a}= {e}^{-\lambda (\frac{{W}_{c}+{L}_{a}}{{V}_{a}}+ \frac{{L}_{c}+{W}_{a}}{{V}_{c}})}$$

where *W*_*c*_, *L*_*c*_ and *V*_*c*_ are the width and length (in m), and speed of vehicles (in m/s), *W*_*a*_, *L*_*a*_ and *V*_*a*_ are the width and length (in m), and speed of animals (in m/s), and ‘λ’ is the traffic volume (in vehicles/s).

*W*_*c*_, *L*_*c*_ and *V*_*c*_ (vehicle characteristics) were calculated for different traffic heterogeneity scenarios, and *W*_*a*_, *L*_*a*_ and *V*_*a*_ (animal characteristics) were calculated for different study species.

The probability of hit or AVC probability (*P*_*h*_) was then calculated as

2 $${P}_{h}=1-{P}_{a}.$$

The model assumes that traversing of roads by animals is ‘blind’ for animals in that they do not respond to the presence of vehicles (i.e. do not stop or turn around), and for drivers in that they do not respond to the presence of animals on the road by braking. Therefore, we define *P*_*h*_ as the probability of hit of an animal that is attempting to cross the road, as we cannot account for the number of crossing attempts that translate into actual presence of animal on the road for an AVC to occur.

Sensitivity of *P*_*h*_ to change in model parameters viz., traffic (vehicle length, vehicle width, vehicle speed and hourly volume) and animal characteristics (body length, width, speed and average group size) was calculated using package “pse”^44^ in R version 3.6^45^. Change in AVC probability with unit change in each model parameter was calculated at 95% confidence intervals while keeping other variables at mean values. We used uniform probability distribution for all the variables except length of animals, length of vehicles and velocity of vehicles for which we used normal probability distribution. We used 1000 bootstraps of the Latin hypercube sampling (LHS) to understand the effect of the variables and to estimate the uncertainty for evaluation of the confidence interval and partial inclination coefficients (PIC). Confidence interval non-overlapping zero signifies a considerable effect of the variable and the sign of the PIC denotes the direction of the effect of the variable.

### Road and traffic characteristics

We obtained traffic volume data for NH 44 from the regional Project Implementation Unit of the National Highway Authority of India for a period of 2 weeks during the study, and extracted the daily and hourly traffic patterns and heterogeneity viz., car/van, LCV (light commercial vehicle), bus/truck, MAV (multi-axle vehicle) and OSV (off-shore vehicle).

Traffic flow speeds were simulated at different hourly traffic volumes (100–2400 vehicles per hour) for present traffic heterogeneity using the traffic-simulation software VISSIM 11.00 Student Edition^46^. We simulated traffic flow on a road section or ‘link’ of 1 km on the VISSIM interface, and specified the width, number of lanes and direction of traffic flow. We set the road type as ‘Freeway (free lane selection)’, and number of lanes at 2 for 4-lane road, and 1 for 2-lane road. For 4-lane road with a median, we generated average speed of vehicles on one side (2-lanes). We followed the same approach for 2-lane road, assuming that overtaking on undivided road did not take place. We set lane widths at 3.5 m^47^, and vehicle composition or heterogeneity as containing three major types of vehicles—car (categories car and LCVs were merged), bus/truck, and MAV (categories MAV and OSV were merged). The headways of the heterogeneous traffic flow were assumed to follow negative exponential distribution^48^, and the free flow speeds of different vehicle types was assumed to follow normal distribution^49^. All model parameters viz., driving behaviour characteristics, vehicle length and width and desired free-flow speed for each vehicle type (following Bains, Ponnu and Arkatkar^50^), and relative flow (proportion of vehicle type in traffic flow or heterogeneity) have been detailed in Supplementary Table S1.

We placed vehicle input points at the beginning of the link, and data collection points midway along the road, and specified the hourly traffic volume at each vehicle input point. The average harmonic speed of traffic flow at each traffic volume and free flow speeds of different traffic components were selected as data collection measurement attributes (outputs of the simulation). Each simulation was run for 600 s (10 min) with 20 replicates. Simulation resolution of 10 time steps/second was set to maximize speed data collection at data collection points.

### Species characteristics

We obtained data on species morphometry viz., length, width^51^, and average group size^51–55^ for the six study species (chital, gaur, leopard, sambar, tiger and wild pig) (Table 1). To account for collective risk for group living species, the average group size was factored into the average length of animal^23^ in Eq. (1).

**Table 1:** Species characteristics including morphometric data and behaviour used for calculation of animal vehicle collision probabilities.

Animal species	Average weight (kg)	Body length (max) (m)	Body width (m)	Mean group size	Maximum running speed (m/s)	Traversing speed (m/s)
Chital *Axis axis*	65	1.55	0.6	8.15	17.49	2.91
Gaur *Bos gaurus*	825	3.3	1	4.75	13.14	2.19
Leopard *Panthera pardus*	49.25	2.43	0.4	1	17.17	2.86
Sambar *Rusa unicolor*	202.5	2.1	0.8	2.85	16.82	2.80
Tiger *Panthera tigris*	173.75	3.1	0.6	1	17.08	2.84
Wild pig *Sus scrofa*	90	2	0.5	5.87	17.6	2.93

We calculated the maximum animal speeds from the equation for terrestrial animals (running) given by Hirt, Jetz, Rall and Brose^56^, based on average animal body weights ‘M’.

3 $${V}_{max}=25.5{M}^{0.26}(1-{e}^{{-22M}^{-0.6}})$$

During the study period, we recorded the walking speeds (in m/s) of the study animals in their natural habitats inside PTR, Maharashtra, to calculate the average moving speed. For each species, we averaged 14–20 observations of various walking speeds which was found to be close to 1/6th of the top running speed (Supplementary Table S2).

### Influence of road and traffic characteristics on AVC probability

With respect to different traffic compositions in a traffic flow (in terms of types of vehicles) specified in Supplementary Table S1, traffic flow speeds for different heterogeneity scenarios namely H_1_–H_9_ were simulated using VISSIM for hourly traffic volumes ranging from 100 to 2400 vehicles per hour (as defined in Section A of Methods under ‘Road and traffic characteristics’).

We calculated AVC probability (*P*_*h*_) under different scenarios of traffic heterogeneity on 2-lane and 4-lane sections of the road using Eq. (1). The exposure risk in seconds i.e., the time on the road for which an animal would be vulnerable to collision as a function of road width and *P*_*h*_ was also calculated.

### Influence of animal activity on AVC risk

The behaviour of animals, in terms of activity patterns in the vicinity of roads, was incorporated into the model in the form of AVC risk, which is the AVC probability under a specific hourly traffic volume for a species, multiplied by the activity (hourly detection probability) of the animal near the road.

To quantify animal activity, we deployed motion-activated ScoutGuard Long Range Incandescent Flash Trail Cameras (https://www.scoutguard.com.au/) in two forest segments (8 × 5 sq. km each) along NH 44 in PTR, Maharashtra and PTR, Madhya Pradesh (Fig. 8). The selected forest segments were intersected by the highway—a 5 km long 2-lane section through PTR, Madhya Pradesh, and a 5 km long 4-lane section through PTR, Maharashtra, Both forest segments were divided into 400 × 400 m grids on both sides of the road, and were similar in terms of vegetation and habitat type, hourly traffic volume, traffic load, and animal densities. Single-sided camera traps were deployed along forest trails and dirt roads in both sections in two phases during July–August 2017, at increasing distances from the highway. The hourly capture rates of animals calculated from camera traps within 0–400 m from the road (n = 47) were used to calculate the roadkill risk under present traffic heterogeneity conditions, assuming that animals captured at this distance were most likely to encounter roads. Roadkill risk was calculated by multiplying hourly capture rates, an indicator of time spent close to road (activity), with the probability of being hit by a vehicle (AVC probability or *P*_*h*_). Overlap of animal and traffic activity was calculated using functions ‘overlapEst’ and ‘bootEst’ in package ‘overlap’^57^ in R version 3.6^45^.

## Supplementary information

Supplementary Information
