# Supplementary material for: Integrating large mammal behaviour and traffic flow to determine traversability of roads with heterogeneous traffic on a Central Indian Highway
Source: Sci Rep. 2020 Nov 3;10:18888. doi: 10.1038/s41598-020-75810-2 (PMC7642331; doi:10.1038/s41598-020-75810-2)
Supplement: Supplementary file 1 — Supplementary Information [file 41598_2020_75810_MOESM1_ESM.docx]

**TITLE**: Integrating Large Mammal Behaviour and Traffic Flow to Determine Traversability of Roads with Heterogeneous Traffic on a Central Indian Highway

**AUTHORS**: Akanksha Saxena^1^, Nilanjan Chatterjee^1^, Asha Rajvanshi^1^, Bilal Habib^1^*

**Supplementary Table S1.** Vehicle and flow characteristics pertaining to present (H_0_) and simulated traffic scenarios (H_1_-H_9_) on NH 44 for simulation of traffic flow in VISSIM. Average car widths and lengths (for calculation of AVC probability using Equation 1) were calculated using relative proportions (flows) of different vehicle types.

| Heterogeneity scenarios | Relative flows (% of vehicles) | | | Average car width (m) | Average car length  (m) |
| --- | --- | --- | --- | --- | --- |
|  | Car | Bus/truck | MAV |  |  |
| H_0_ | 61 | 13 | 26 | 2.073 | 5.21 |
| H_1_ | 100 | 0 | 0 | 1.8 | 3 |
| H_2_ | 0 | 100 | 0 | 2.5 | 6 |
| H_3_ | 0 | 0 | 100 | 2.5 | 10 |
| H_4_ | 75 | 25 | 0 | 1.975 | 3.75 |
| H_5_ | 0 | 75 | 25 | 2.5 | 7 |
| H_6_ | 12.5 | 12.5 | 75 | 2.4125 | 8.625 |
| H_7_ | 50 | 25 | 25 | 2.15 | 5.5 |
| H_8_ | 25 | 50 | 25 | 2.325 | 6.25 |
| H_9_ | 25 | 25 | 50 | 2.325 | 7.25 |
|  | | | | | |
| Vehicle traits |  |  |  |  |  |
| Width (m) | 1.8 | 2.5 | 2.5 |  |  |
| Length (m) | 3 | 6 | 10 |  |  |
| Speed distribution (m/s) | 62-100 | 49-93 | 40-62 |  |  |

**Supplementary Table S2**. Average movement speeds of animals based on field observations of animal movement within forests for 50 m segments.

| Species | Number of 50 m segments | Total distance (m) | Average time taken (seconds) | Average observed speed (m/s) |
| --- | --- | --- | --- | --- |
| Chital | 20 | 950 | 16.66 | 3 |
| Gaur | 18 | 900 | 25 | 2 |
| Leopard | 14 | 700 | 18.51 | 2.7 |
| Sambar | 20 | 1000 | 18.51 | 2.7 |
| Tiger | 17 | 850 | 17.24 | 2.9 |
| Wild pig | 20 | 1000 | 16.66 | 3 |

**Supplementary Table S3.** AVC risk at peak traffic (1700 hrs) on NH 7 and animal activity hours. Activity peaks of animals pertain to peak activity at a distance of 0-400 m from road. Chital has two peak hours of activity near the road (0700 and 1900 hours), and therefore has two values for AVC-risk on 2-lane and 4-lane each.

| Species | Peak  animal activity time (h) | AVC risk on 2-lane at | | AVC risk on 4-lane at | |
| --- | --- | --- | --- | --- | --- |
|  |  | Peak traffic | Peak animal activity | Peak traffic | Peak animal activity |
| Chital | 0700 | 0.02 | 0.015 | 0.02 | 0.01 |
|  | 1900 |  | 0.03 |  | 0.03 |
| Gaur | 2100 | 0 | 0.10 | 0 | 0.10 |
| Sambar | 2000 | 0 | 0.03 | 0 | 0.03 |
| Wild pig | 1900 | 0.03 | 0.05 | 0.03 | 0.05 |

**Supplementary Table S4.** Activity overlap coefficient estimates between traffic and different study species.

| Species | Overlap Coefficient (Dhat) | Bootstrapped Dhat | C.I. |
| --- | --- | --- | --- |
| Chital | Dhat4 = 0.80 | 0.82 | 0.77 - 0.83 |
| Sambar | Dhat1 = 0.64 | 0.62 | 0.49 - 0.79 |
| Gaur | Dhat1 = 0.49 | 0.52 | 0.38 - 0.59 |
| Wild pig | Dhat4 = 0.81 | 0.82 | 0.73 - 0.89 |

**
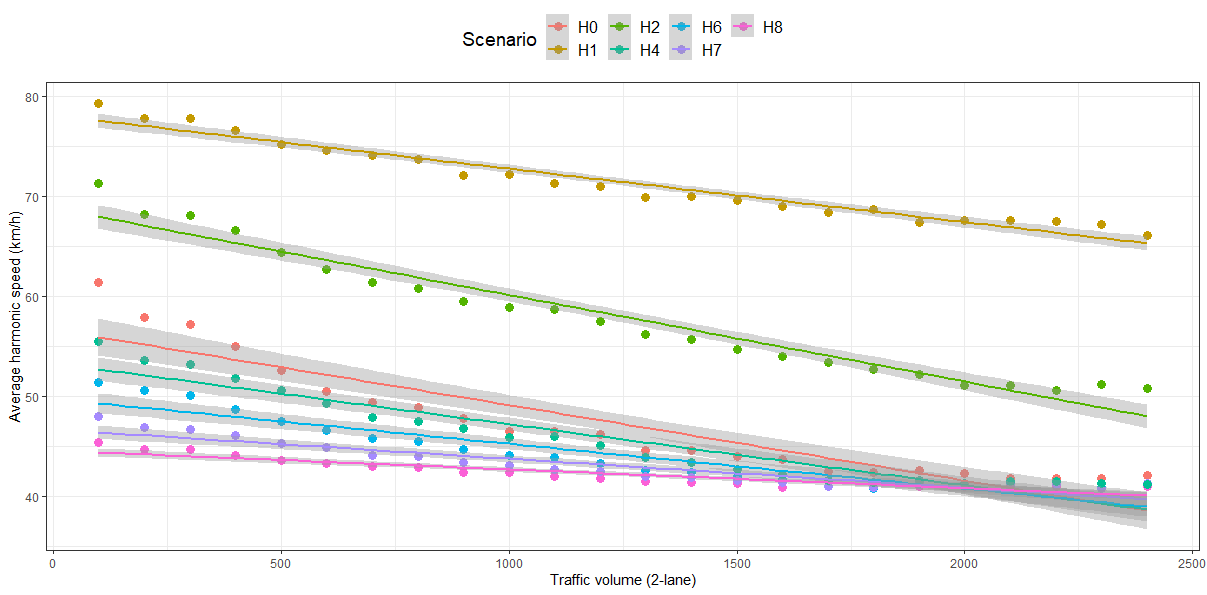
**

**
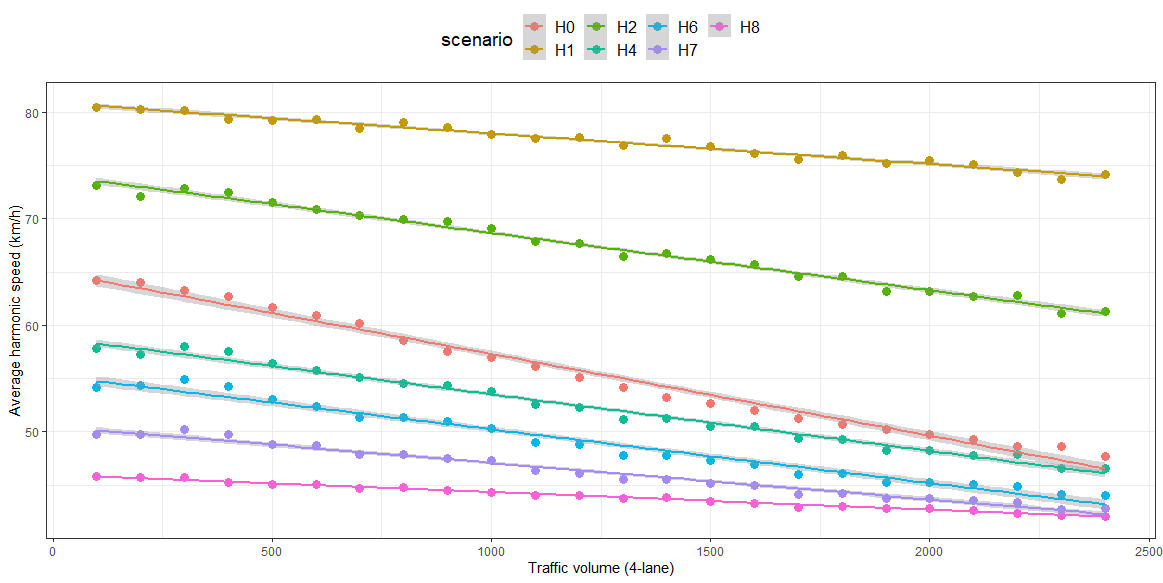
**

**Supplementary Figure S1.** Traffic flow speed distributions for different traffic heterogeneity scenarios with increasing traffic volumes on (a) 2-lane and (b) 4-lane of National Highway 44, Pench Tiger Reserve, India


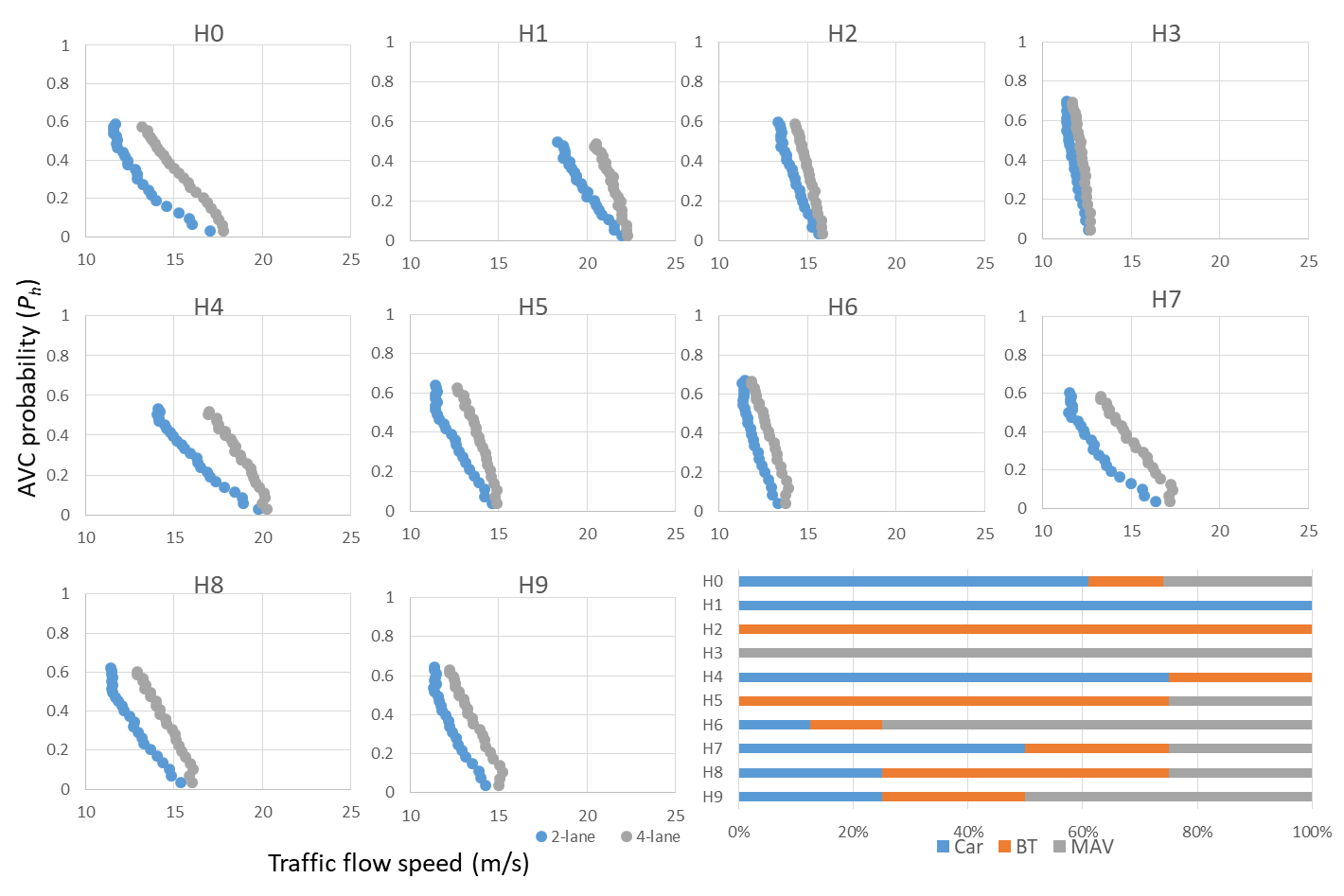


**Supplementary Figure S2.** Variation in *P_h_* with traffic flow speeds under different traffic heterogeneity scenarios on 2-lane and 4-lane road segments. The traffic flow speed for traffic scenarios with predominantly heavy vehicles (H_2_, H_3_, H_6_) decreased at a lower rate (9.58% on 2-lane and 8.18% on 4-lane) with increasing traffic volume, thereby reducing the rate at which *P_h_* increased. Conversely, traffic flow speed for traffic comprising mostly of light vehicles (H_0_, H_4_, H_7_) decreased at a higher rate with increasing traffic volume, thus showing a rapid increase in *P_h_*. Change in traffic flow speed was highest for heterogeneity scenarios with mixed traffic composition (31.41% on 2-lane and 25.78% on 4-lane).
